# Author Correction: Combination of sphingosine-1-phosphate receptor 1 (S1PR1) agonist and antiviral drug: a potential therapy against pathogenic influenza virus

**DOI:** 10.1038/s41598-020-69835-w

**Published:** 2020-07-28

**Authors:** Jiangnan Zhao, Meiying Zhu, Hao Jiang, Simen Shen, Xin Su, Yi Shi

**Affiliations:** 1 Department of Respiratory and Critical Medicine, Jinling Hospital, Nanjing University School of Medicine, Nanjing, 210002 China; 2 Department of Emergency Medicine, the Second Affiliated Hospital, Southeast University, Nanjing, 210002 China; 3 Department of Respiratory Medicine, the First People’s Hospital of Nantong, Nantong, 226000 China

Correction to: *Scientific Reports* 10.1038/s41598-019-41760-7, published online 27 March 2019

This Article contains an error in Panel D of Figure 3. The third pathological image (S1PR1 ECKO + Vehicle) is a duplicate of the fourth pathological image (S1PR1 ECKO + CYM-5442), and therefore incorrect. The correct version of Figure 3 is reproduced below.

**Figure 3:**
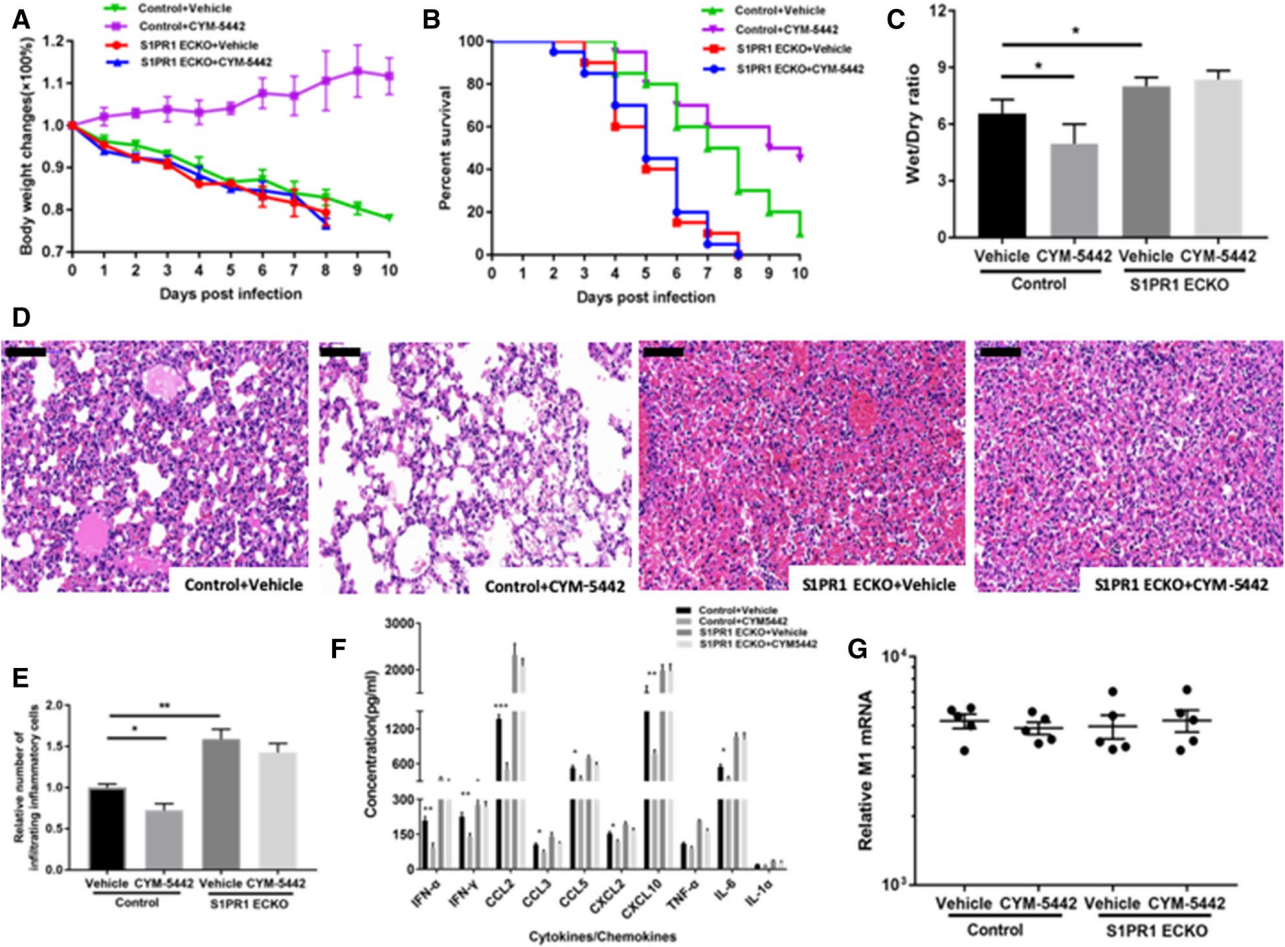
Administration of CYM-5442 ameliorates ALI induced by H1N1 virus infection in control mice, but no effect was observed on* S1PR1 ECKO* mice. Control and *S1PR1 ECKO* mice were infected with 10^5.5^ TCID50 of influenza A virus and treated with either vehicle or CYM-5442.** (A)** Body weight change curves (n = 20). The differences between vehicle and CYM-5442-treated control mice were significant (P < 0.001).** (B)** Kaplan-Meier survival curves (n = 20). Control mice treated with CYM-5442 survived longer than untreated control mice (P = 0.033).** (C)** Wet/dry ratios of lungs from infected mice (n = 6).** (D)** Representative pathological images of the lungs on day 5 post-infection. Lung injury was significantly less in control mice receiving CYM-5442 with less pulmonary consolidation, exudation in alveolar air space, vascular haemorrhage and inflammatory infiltrates than lung injury in vehicle recipients. Magnification: 200× ; scale bars, 100 μm.** (E)** Relative average quantification of inflammatory cells in lung tissues on day 5 post-infection (n = 5).** (F)** Concentrations of cytokines/chemokines were measured at 48 hours post-infection by ELISA in BALF (n = 5).** (G)** Viral loads of 2009 H1N1 in lung tissues on day 3 post-infection (n = 5). Using uninfected mice lung RNA as a baseline, equivalent levels of influenza virus RNA occurred in all four groups. *P < 0.05, **P < 0.01 and ***P < 0.001. These data are representative of three experiments and are shown as the means ± SEM.

